# Site-specific phosphorylation of casein kinase 1 δ (CK1δ) regulates its activity towards the circadian regulator PER2

**DOI:** 10.1371/journal.pone.0177834

**Published:** 2017-05-17

**Authors:** Gracie Wee Ling Eng, Edison, David M. Virshup

**Affiliations:** 1 Programme in Cancer and Stem Cell Biology, Duke-NUS Medical School, Singapore, Singapore; 2 Department of Pediatrics, Duke University School of Medicine, Durham, North Carolina, United States of America; McGill University, CANADA

## Abstract

Circadian rhythms are intrinsic ~24 hour cycles that regulate diverse aspects of physiology, and in turn are regulated by interactions with the external environment. Casein kinase 1 delta (CK1δ, CSNK1D) is a key regulator of the clock, phosphorylating both stabilizing and destabilizing sites on the PER2 protein, in a mechanism known as the phosphoswitch. CK1δ can itself be regulated by phosphorylation on its regulatory domain, but the specific sites involved, and the role this plays in control of circadian rhythms as well as other CK1-dependent processes is not well understood. Using a sensitized PER2::LUC reporter assay, we identified a specific phosphorylation site, T347, on CK1δ, that regulates CK1δ activity towards PER2. A mutant CK1δ T347A was more active in promoting PER2 degradation. This CK1δ regulatory site is phosphorylated in cells in trans by dinaciclib- and staurosporine-sensitive kinases, consistent with their potential regulation by cyclin dependent and other proline-directed kinases. The regulation of CK1δ by site-specific phosphorylation via the cell cycle and other signaling pathways provides a mechanism to couple external stimuli to regulation of CK1δ-dependent pathways including the circadian clock.

## Introduction

Circadian rhythms are intrinsic ~24 hour cycles of behavioral, neural, hormonal and biochemical processes occurring in most organisms exposed to daily changes in light and dark. These rhythms are controlled by a master clock in the suprachiasmatic nucleus (SCN) of the hypothalamus, which in turn can be reset by external light cues via input from ganglion cells in the retina. The master clock synchronizes intrinsic clocks present in virtually all cells and tissues throughout the body and can couple to the cell cycle in various tissues [1,2]. Circadian rhythms in diverse tissues coordinate tissue specific functions such as digestion, sleep, and motor activity. Disruption of circadian rhythms, for example by shift work, jet lag, or sleep deprivation increases the risk of multiple diseases including diabetes, heart disease, mood disorders, and cancer [3–7].

The vertebrate circadian clock has at its core coupled transcriptional-translational-degradation feedback loops that have been extensively studied and reviewed [8–10]. Clock and BMAL1 are positively acting transcription factors regulating the expression of diverse genes, including *Period* (*PER*) and *Cryptochrome* (*CRY*). PER and CRY proteins are in turn repressors of Clock/BMAL1 activity. PER and CRY proteins accumulate throughout the prospective day, peaking in the early night to turn off their own transcription. PER1 and PER2 undergo multisite phosphorylation by Casein kinase 1 δ and ε (CK1δ and CK1ε) and are degraded, resulting in a trough in protein level in the early day[11–14]. The repression of Clock/BMAL1 is thus lifted, and a new cycle starts early in the prospective day.

The biochemical role of casein kinases 1 δ and ε (CK1δ and ε) in regulating PER stability is becoming clearer. Phosphorylation of PER1 and PER2 at amino acids 478 on mPER2 by CK1δ/ε creates a recognition site for β-TrCP binding [13,15]. β-TrCP is an F-box protein that is the adaptor subunit of the SCF (Skp1-Cullin-F-box) E3 ubiquitin ligase complex [16]. Upon β-TrCP binding, phosphorylated PER is ubiquitylated and degraded via the proteasomal pathway. Degradation of PER2 is accelerated in individuals with Familial Advanced Sleep Phase (FASP), due to a PER2 S662G missense mutation [17]. Phosphorylation of the wild-type FASP site by an unidentified priming kinase, followed by downstream phosphorylation of up to 4 additional serines by CK1 was demonstrated to increase PER2 abundance [18,19]. We recently demonstrated that phosphorylation of the FASP region (S662 in humans, S659 in mice and downstream sites) prevents CK1 phosphorylation of the β-TrCP (S478) site, explaining why FASP phosphorylation is a stabilizing event and why mutation of the priming site destabilizes PER2 [11].

Because of its key role in PER phosphorylation and the circadian clock, the regulation of CK1 is of great interest. CK1δ and ε are rapidly and reversibly heavily phosphorylated in vivo and this phosphorylation regulates the activity of the kinases [20–22]. A number of these *in vivo* phosphorylation sites in the CK1δ and CK1ε carboxyl-terminal tail result from intra-molecular autophosphorylation [20]. Inhibiting CK1δ/ε kinase activity with small molecules such as PF670462 (which inhibits both isoforms) or PF4800567 (which is CK1ε-specific) can prevent autophosphorylation, but not phosphorylation in trans by other kinases such as CDK5, PKA, and CHK1 [23–25]. These inhibitory phosphorylation sites turn over rapidly, as *in vivo* hyperphosphorylation of CK1δ and CK1ε can be induced by short exposure to phosphatase inhibitors such as calyculin A [20,21,26]. Removal of the inhibitory phosphorylation sites by limited proteolysis or truncation of the tail, or mutation of multiple serine and threonine to alanine residues at specific sites resulted in increased activity of CK1ε against its substrates in *in vitro* assays [20,21,27,28]. These inhibitory phosphorylation sites on CK1δ and CK1ε can be regulated in vivo by signaling via metabotropic glutamate receptors, Wnts, and cyclin dependent kinases, and presumably by additional pathways as well [23,29,30]. However, specific physiologically important phosphorylation sites on CK1δ and CK1ε have not yet been identified.

CK1δ is the key regulator of circadian rhythms *in vivo* [31,32]. We hypothesized that the phosphorylation status of the CK1δ autoregulatory domain plays a role in the regulation of circadian rhythms. We established a sensitized assay where PER2 stability is exquisitely sensitive to CK1δ activity. A multi-phosphorylation site mutant of CK1δ showed increased specific activity that accelerated PER2 degradation. CK1δ T347 was identified as a key phosphorylation site regulating PER2 stability. We generated a phosphoepitope-specific antibody, and found that CK1δ T347 phosphorylation is not due to autophosphorylation, but rather is targeted by multiple kinases including cyclin-dependent kinases. Inhibition of T347 phosphorylation decreased the stability of PER2. Taken together, these data show that CK1 regulation of PER stability can be influenced by additional intracellular kinases impinging on the phosphorylation of CK1δ T347, providing a pathway for extracellular and intracellular stimuli to influence circadian rhythms.

## Materials and methods

### Reagents

pCK1δ plasmids were pCS2-6Myc-CK1δ (V2418). PF670462, PF4800567 and staurosporine were purchased from Tocris Bioscience. Dephosphorylated casein was purchased from Sigma Aldrich. Cell lines were from American Type Culture Collection (ATCC), USA.

### Antibodies

Commercial antibodies were sourced as follows: firefly luciferase Ab (Abcam ab21176), Myc mAb (4A6, Millipore 05–724), CK1δ mAb (AF12G4, Abcam ab85320), β-actin Ab (Cell Signaling #4967), β-tubulin Ab (EP1569Y, Abcam ab52623). Phospho-S478 PER2 [11], and PP2A c-subunit Ab (109–4) were described previously.

### Cell culture conditions

Cells were cultured in DMEM (Nacalai Tesque) in the presence of 10% FBS (Gibco), 1x Pen/Strep (Gibco) and 1x Sodium Pyruvate (Gibco), in a humidified incubator conditions at 37°C with 5% CO_2_, unless otherwise stated. For transfection, cells were seeded 1 day pre-transfection to achieve 70% confluency on day of transfection. Cells were transfected with TurboFect (Fermentas) with the indicated amounts of DNA according to manufacturer’s manual.

### PER2 half-life measurement by LumiCycle

PER2::LUC expression plasmids were transiently transfected alone or with CK1δ/ε in 35mm dishes, in phenol red-free DMEM, in the presence of 0.1 M D-luciferin, 10 mM Hepes and 1.2 g/L sodium bicarbonate. Dishes were sealed with 40 mm cover glasses and vacuum grease, and incubated in the LumiCycle. The next day, 40 μg/ml cycloheximide (Sigma) was added per dish. Luminescence data were used to calculate PER2 half-life in Graphpad Prism using one-phase decay algorithm as described previously[11]. PER2 half-life was measured in unsynchronized cells. Synchronization was avoided as it would introduce additional variability, since over-expression of wildtype or mutant PER2 and CK1δ would alter the timing of the cells in each experiment.

### SDS-PAGE and western blot

Cells were washed once in 1x PBS and lysed in either 4% SDS or Hepes Lysis Buffer (Hepes pH 7.5, NaCl, EDTA, 0.1% NP-40, 1x protease inhibitor cocktail). SDS lysates were boiled for 5 minutes before being mechanically sheared by syringe and 27½G needle. NP-40 lysates were centrifuged at 13,000 rpm, 4°C for 10 minutes to remove cell debris. Protein quantification was performed using BCA quantification (Pierce #23227). 20–50 μg lysates were added to 4x sample buffer and resolved on SDS-PAGE. Protein ladders used were Bio-Rad’s All Blue (#161–0373) or WesternC (#161–0376) Standards.

SDS-PAGE gels were 10% unless otherwise stated. Resolved lysates were transferred to PVDF membranes (Immobilon IPFL00010) in a wet transfer system in transfer buffer (25 mM Tris, 184 mM Glycine, 20% Methanol, 0.01% SDS). Membranes were then blocked in 5% BSA. Depending on the antibodies, visualization was performed on either by fluorescence on Licor-Odyssey, or by enhanced chemiluminescence on ImageQuant (GE Healthcare) using Immunestar or Clarity (Bio-Rad 170–5061) ECL reagent.

### Generation of non-phosphorylatable mutants

Putative *in vivo* phosphorylation sites on CK1δ/ε were identified from the PhosphoSite Plus database on 1 December, 2015 (www.phosphosite.org). cDNA sequences of mutant carboxyl-terminal tails were synthesized by GeneScript in a pUC57 backbone, and used to replace CK1δ wildtype sequence by EcoRV blunt ligation. cDNA were PCR amplified with primers N1073 (CCGGCAGGGCTTCTCA|TATGACTACGTGTTCGACTG) and N1075 (CATTCCACAGGGTCGA|CCACTGTGCTGGCGAATT) using Platinum Taq kit. pBABE-6Myc-CK1δ (V1250) was engineered to carry an internal NdeI cut site using site-directed mutagenesis. These were then linearized using NdeI and SalI. Linearized backbone and PCR products were resolved on agarose gel electrophoresis, excised, and purified using QiaQuick gel extraction kit. PCR products were then cloned into linearized backbones using ColdFusion cloning kit (SBI MC101B-1), at a 2:1 insert:vector molar ratio.

Constructs for transient transfections were made by cloning from the pBABE constructs into pCS2 vector. The pBABE constructs were digested with ClaI and AvrII. pCS2-EV (V513) was digested with ClaI and XbaI. Digested products were resolved on agarose gel electrophoresis. Bands of interest were then excised and purified using MN NucleoSpin Extract II kit. Ligation of vector and insert was performed using Fermentas Rapid Ligation Kit.

### In vitro immunoprecipitation(IP)-kinase assay

Cells were lysed in kinase assay lysis buffer (50 mM Hepes (pH 7.5), 1% NP-40, 0.1% SDS, 150 mM NaCl, 20 mM β-glycerol phosphate, 1mM DTT and 1x protease inhibitor cocktail), and centrifuged 13, 000 rpm, 4°C, 15 minutes. Immunoprecipitation was performed using the stated antibody with overnight tumbling at 4°C. Protein A/G beads slurry was added the next morning, and tumbled a further 2 hours at 4°C. Beads were pelleted at 1,500 rpm for 5 minutes. Beads were then washed once with kinase assay lysis buffer, once with kinase assay lysis buffer without SDS and DTT, and twice with CK1 storage buffer (10% sucrose, 30 mM Hepes (pH 7.5), 15 mM NaCl, 1 mM DTT). Beads were split into 3 fractions at the final wash step before buffer removal.

Substrate mixture (20 mM Tris-Cl (pH 8.0), 5 mM MgCl_2_, 0.5 mM DTT, 150 mM KCl, 10 μg dephosphorylated casein (Sigma), 40 nM Calyculin A) was added to one set of beads and incubated on ice for 5 minutes. ATP mix (2 μCi ^32^P-ATP (Perkin Elmer #BLU502A250UC), 100 μM ATP) was then added to the beads and incubated at 37°C for 15 minutes. To pre-autophosphorylate, ATP mix and reaction buffer with casein omitted was added to a second set of beads and pre-incubated at 37°C for 15 minutes, before the addition of casein followed by an additional incubation at 37°C for 15 minutes. All reactions were stopped by the addition of 6x SDS-sample buffer. The samples are resolved on 4–20% gradient SDS-PAGE (Biorad), the gel stained with Coomassie Blue (Expedion Instant Blue ISB1L), and exposed to a Phosphor Screen overnight. Screen is imaged on the Typhoon FLA9500 (GE Healthcare 28-9969-43). One set of beads were used for western blot to check for equal immunoprecipitation and loading.

### Site directed mutagenesis

Primers were designed using Stratagene’s primer design software. 20 ng template DNA was used for site-directed mutagenesis with Pfu Ultra (Stratagene), according to Quikchange protocol. Methylated DNA was digested with DpnI (NEB) at 37°C for 2 hours. 2 μl of the reaction mix was transformed into Top10 cells. Plasmids were extracted using Machery-Nagel miniprep kit.

### Immunoprecipitation

Cells were washed once with 1x PBS, and lysed in HTS buffer (100 mM Hepes pH7.5, 150 mM NaCl, 1% Triton X-100, 2mM DTT, 1x protease inhibitor cocktail (Roche)). Lysates were centrifuged at 13,000 rpm at 4°C, for 10 minutes to remove cell debris. Supernatants were collected and pre-cleared with 10 μl of Protein A/G beads slurry with tumbling for 30 minutes at 4°C. Protein quantification was performed using Bradford’s assay (Bio-Rad #500–0002). 500 μg protein were immunoprecipitated with 1 μg antibodies indicated, unless otherwise stated. Immune-complexes were allowed to tumble overnight at 4°C. Following morning, 20 μl agarose Protein A/G were added. Immune-complexes were allowed to tumble at 4°C for 2 hour. Beads were washed 3 times with 1 ml lysis buffer without DTT and protease inhibitor. Elution was performed using 2x sample buffer and boiled at 95°C for 5 min.

### Statistical analyses

All statistical analyses were done using Graphpad Prism. Half-lives were calculated using the one-phase decay algorithm using the luciferase activity, from the point of cycloheximide addition to the plateau at minimum luciferase activity. All half-life calculations shown are of three independent experiments done in duplicates. Adjusted p-values were calculated using 1- or 2-way ANOVA with Tukey’s post-hoc correction using GraphPad Prism. In all figures, * indicates p<0.05, ** p<0.01, *** p<0.001, **** p<0.0001.

## Results

### The S659A mutation sensitizes PER2 to CK1δ mediated degradation

**Fig 1:**
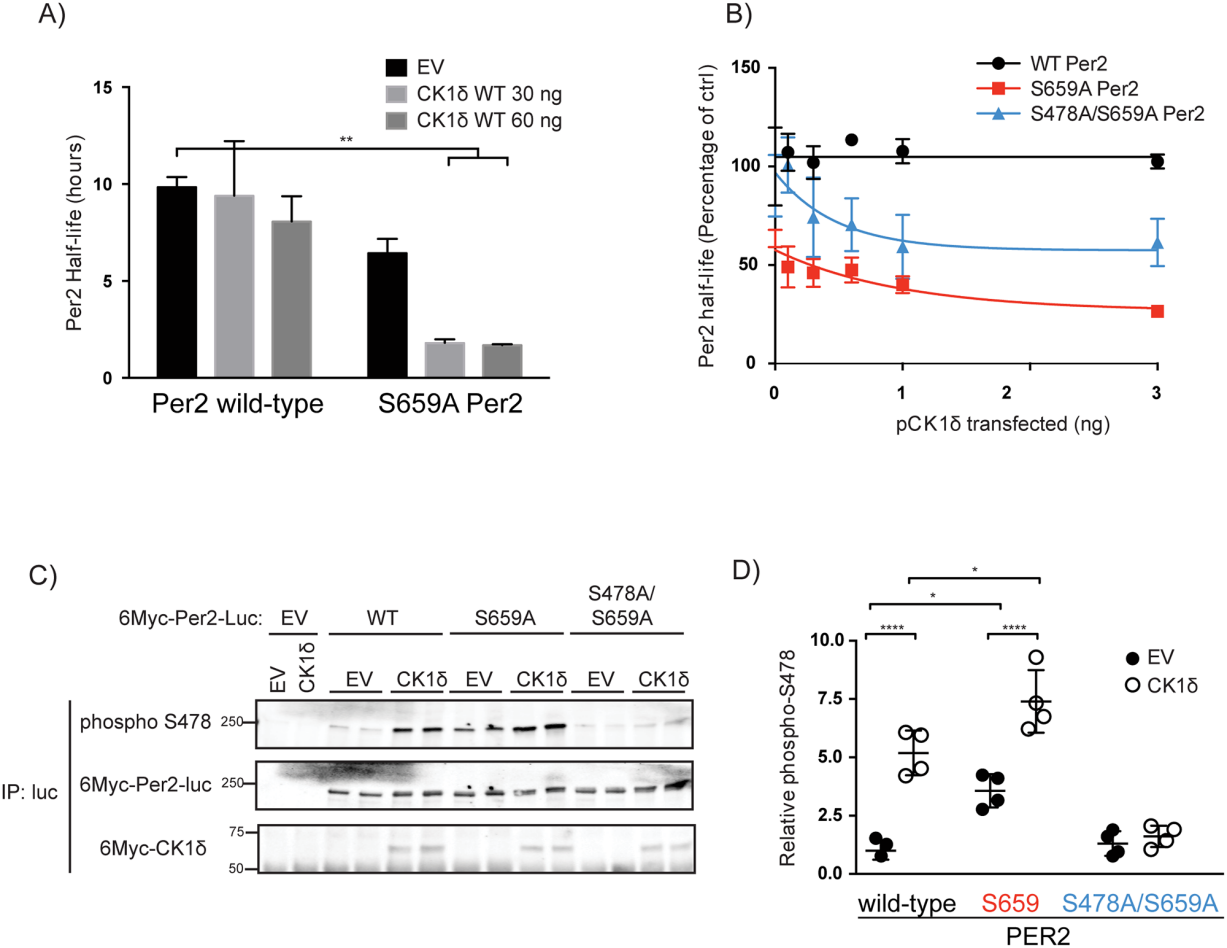
CK1δ mediate increased degradation of PER2(S659A) through phosphorylation at S478. **A) The stability of PER2(S659A) is sensitive to CK1δ activity.** 50 ng of plasmid encoding wild-type or S659A PER2::LUC was co-transfected into HEK293 cells with indicated amount of empty vector (EV), or pCK1δ. Cycloheximide was added 20 hours post-transfection and the half-life of PER2::LUC was calculated using Graphpad Prism’s one-phase decay algorithm. **B) S478A mutation partially rescues PER2(S659A) instability.** 10 ng of wildtype, S659A or S478A/S659A double mutant were co-transfected into HEK293 cells with indicated amount of pCK1δ. Cycloheximide was added 20 hours post-transfection and the half-life of PER2::LUC was calculated as above. Half-life shown is plotted as a percentage, where the half-lives for samples with no pCK1δ co-expressed were set as 100%. **C, D) Mutation of S659 increases phosphorylation of the β-TrCP site in a CK1δ sensitive manner.** 6Myc-tagged PER2::LUC was co-expressed in HEK293 cells with empty vector (EV) or 6Myc-tagged pCK1δ in the presence of β-TrCP(ΔFbox) to prevent PER2 degradation. PER2::LUC was immunoprecipitated using an antibody targeting firefly luciferase, and immunoblotted using the indicated antibodies. Quantification of three independent experiments done in duplicates by densitometry is shown in D, where phospho-S478 is normalized to total PER2.

There are at least two major CK1 phosphorylation sites on PER2 with opposing consequences for PER2 stability. Phosphorylation in the FASP region stabilizes the protein, while phosphorylation at the β-TrCP site destabilizes PER2 [11,13,17–19]. Mutation of S659 to alanine (S659A) in the FASP region on mouse PER2 prevents phosphorylation of the FASP region and exposes the β-TrCP site to CK1, leading to a short half-life mutant PER2 that is sensitive to CK1 activity. A PER2::luciferase (PER2::LUC) fusion protein accurately reports the abundance of PERs *in vivo* [33]. We took advantage of this to generate a PER2::LUC reporter fusion protein sensitive to changes in CK1 activity. The half-life of transiently expressed PER2::LUC was ~10 hours, while the half-life of PER2(S659A)::LUC was ~6 hours (Fig 1A). PER2(S659A)::LUC was also more sensitive to CK1 abundance than was PER2::LUC (Fig 1B).

We have previously shown that mutation of mouse PER2 S659 increased the phosphorylation of PER2 at S478 by CK1ε [11]. To confirm that the increased sensitivity of PER2(S659A) to CK1δ was also due to phosphorylation at S478, we co-expressed PER2 with CK1δ. CK1δ increased phospho-S478 on PER2 (Fig 1C and 1D). We blocked PER2 degradation by including dominant negative β-TrCP [13]. When S659 was mutated, the basal phosphorylation of S478 increased. This phosphorylation S478 was further enhanced by the co-expression of CK1δ, consistent with the shortened half-life.

To test if S478 was the major phosphorylation site driving PER2 degradation, wildtype, S659A or S478A/S659A double mutant PER2::LUC were co-expressed with increasing amounts of CK1δ. Mutation at S478 partially restored PER2(S659A) stability (Fig 1B). This suggests that while the decreased stability of S659A was due to increased phosphorylation of the destabilizing S478 site, there may be additional CK1δ dependent destabilizing sites on PER2.

Taken together, these data demonstrate that PER2(S659A)::LUC provides a sensitized background to investigate the effects of changes in CK1 activity, mediated, at least partially, through increased phosphorylation of PER2 at the destabilizing S478 site.

### CK1δ accelerates S659A PER2::LUC degradation in a dose-dependent manner

**Fig 2:**
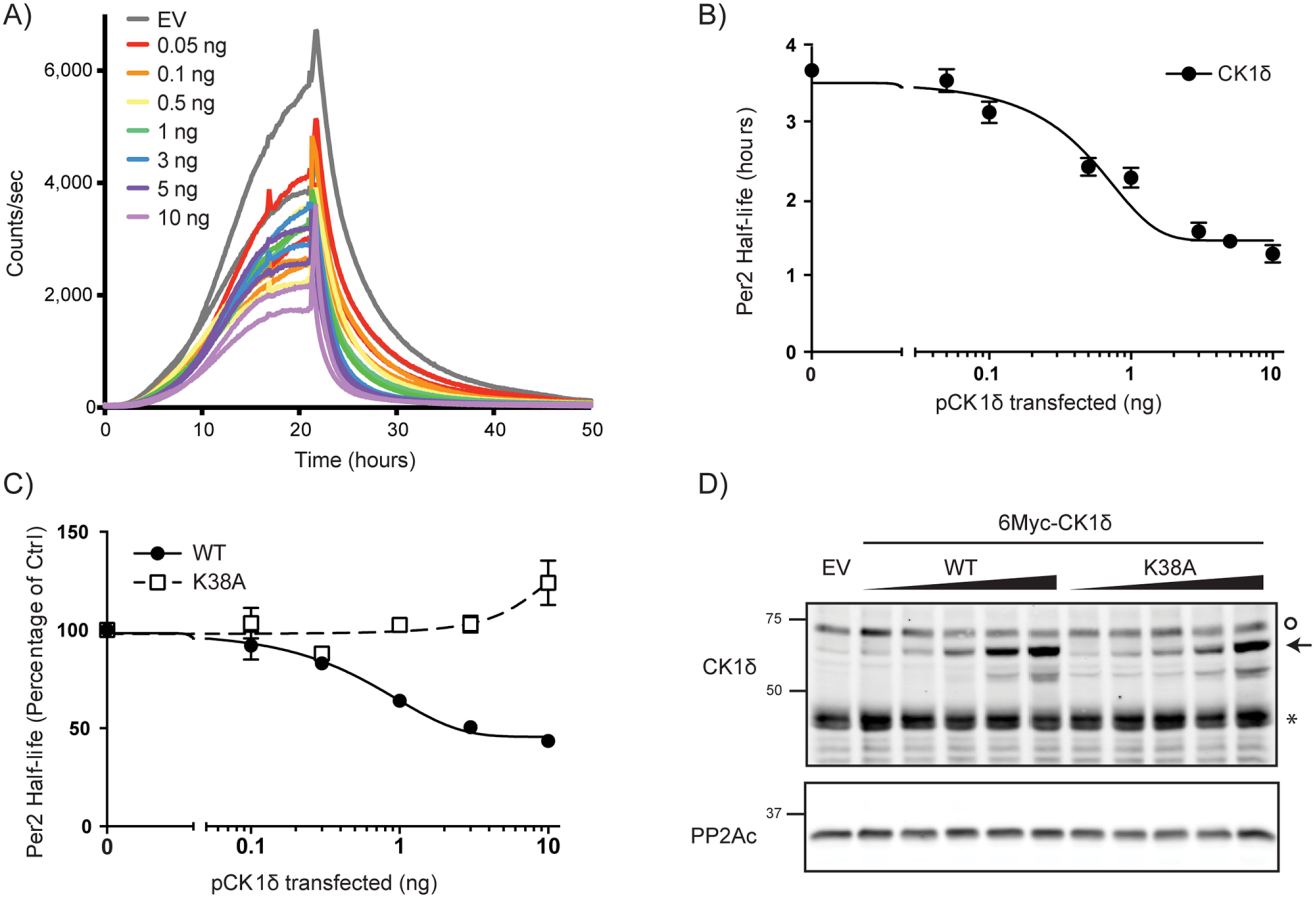
CK1δ accelerates degradation of sensitized PER2. **A, B) Dose-dependent acceleration of S659A Per2 degradation.** S659A PER2::LUC was co-expressed with CK1δ (pCK1δ) in HEK293 cells as above and cycloheximide was added at the 24 hour time point. Increasing amounts of pCK1δ as indicated led to steeper slopes and shorter half-life of PER2 S659A-luc. PER2::LUC half-life is shown in panel B as a function of added CK1δ plasmid. **C-D) CK1δ kinase activity is required to accelerate PER2 degradation**. The half-life of PER2 S659A::LUC was assessed after co-expression in HEK293 cells with wild-type or kinase-dead pCK1δ K38A. **D) Expression of pCK1δ wild-type and K38A** in Fig 2C was confirmed by immunoblot using anti-CK1δ Mab. Arrows indicate Myc-tagged CK1δ while asterisk indicate endogenous CK1δ, and the O indicates a non-specific band. PP2Ac was probed as a load control. Data shown are average from three independent experiments done in duplicates.

To more quantitatively assess the effect of CK1δ activity on PER2(S659A) degradation, picogram and nanogram quantities of CK1δ expression plasmid were co-transfected with only 10 ng of *Per2(S659A)-luc* plasmid (Fig 2A). This amount of *Per2* expression plasmid was chosen as it produced luciferase activity at levels similar to that obtained from endogenously expressed *Per2-luc* knockin in mouse embryo fibroblasts[11,33]. Notably, at this lower amount of *Per2-luc* plasmid, the half-life of S659A PER2::LUC after cycloheximide treatment was significantly shorter (3.7 hrs) than that seen in cells receiving 50 ng plasmid (6.4 hrs). Importantly, increasing amounts of CK1δ expression decreased PER2::LUC half-life in a dose dependent manner, with half-maximal decrease occurring at ~500 pg CK1δ expression plasmid (Fig 2B). Consistent with the presence of both CK1-dependent and independent degradation pathways, CK1 over-expression never shortened the PER2::LUC half-life more than 60%.

To determine if this increase in PER2 degradation was kinase activity dependent, we co-expressed catalytically inactive CK1δ K38A with PER2(S659A)::LUC. Unlike CK1δ, CK1δ(K38A) expression had no effect on PER2 half-life until the highest levels, when the half-life increased, consistent with a dominant negative effect of CK1δ(K38A) (Fig 2C and 2D).

### Phosphorylation site mutant of CK1δ has increased kinase activity in vitro

**Fig 3:**
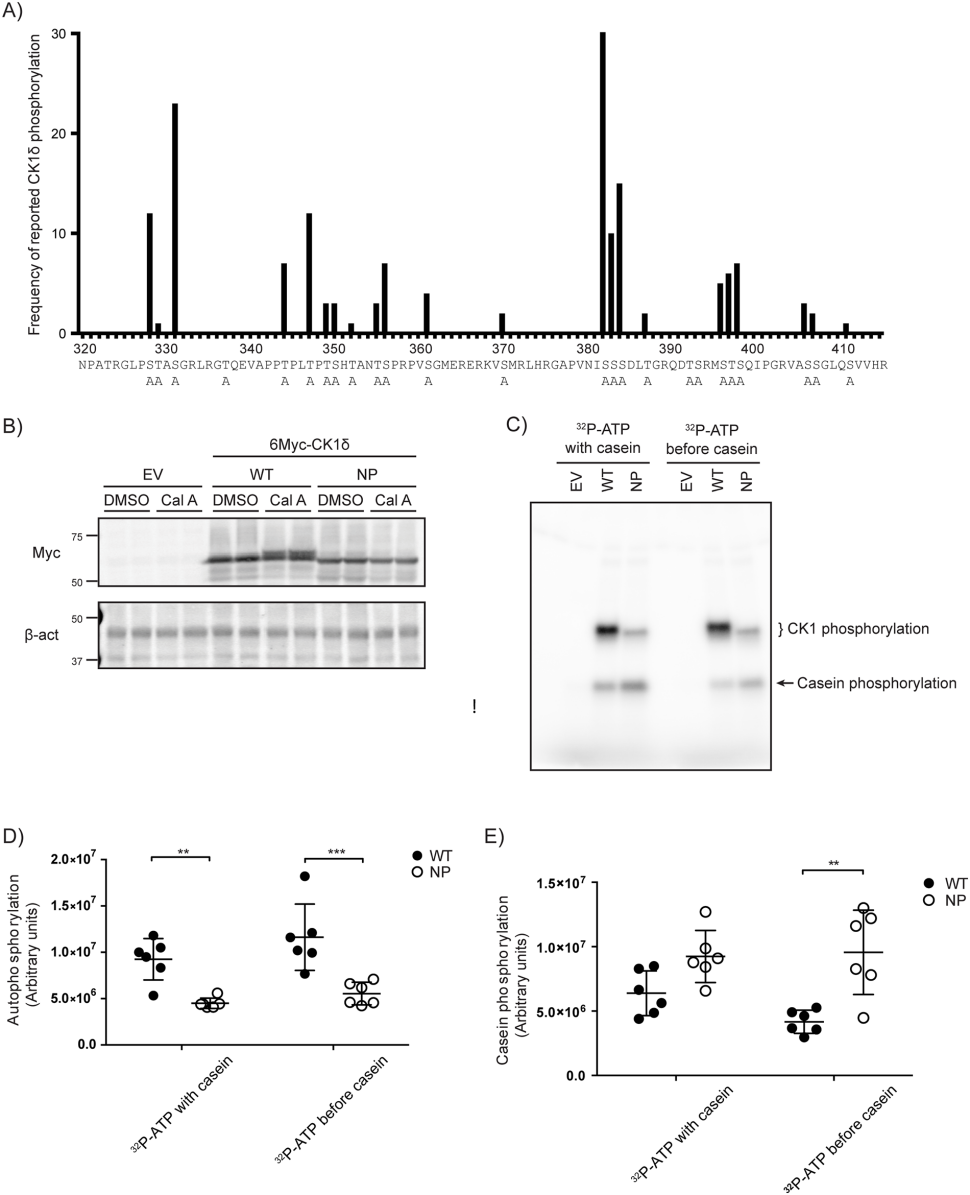
Characterization of non-phosphorylatable CK1δ. **A) Putative CK1δ phosphorylation sites**, based on the number of reports indicating the site is phosphorylated in PhosphoSite Plus database (http://www.phosphosite.org/) as of December, 2015. Sites mutated to alanine in the non-phosphorylatable (NP) mutant CK1δ are indicated by A below the sequence. **B) CK1δ NP mutant does not exhibit a phosphorylation-dependent electrophoretic mobility shift.** pCK1δ WT or NP was transiently expressed in HEK293 cells. 20 hours post-transfection, cells were treated with either DMSO or the serine-threonine phosphatase inhibitor calyculin A (Cal A) (40 nM) for 30 minutes. Lysates were analyzed by immunoblotting with the indicated antibodies. **C-E) CK1δ NP shows decreased autophosphorylation and increased activity *in vitro*.** 6Myc-tagged CK1δ was transiently expressed in HEK293 cells. 20 hours post-transfection, CK1δ was immunoprecipitated and kinase activity assessed using casein as a substrate in a 15 minute reaction (with casein). To assess autophosphorylation, one set of immunoprecipitates were pre-incubated with 100 μM [γ-^32^P]ATP for 15 minutes prior to the addition of casein (before casein). Reactions were stopped by the addition of SDS-PAGE sample buffer and analyzed by SDS-PAGE and PhosphorImager analysis. **C.** Representative autoradiogram of ^32^P-phosphorylated CK1δ and casein. **D, E.** Quantitation of C from three independent experiments done in duplicates.

CK1δ can be phosphorylated on its carboxyl terminal tail and this is inhibitory to its activity [21,22]. Earlier studies of the related kinase, CK1ε identified phosphorylated sites using *in vitro* autophosphorylation of bacterially expressed recombinant protein [28]. In the present study, high confidence *in vivo* phosphorylation sites on the CK1δ carboxyl terminal tail were identified using PhosphoSite Plus [34]. This approach has the advantage of identifying both autophosphorylation sites and sites phosphorylated by other kinases. As a first test of the role of phosphorylation of the CK1δ tail, we generated a non-phosphorylatable mutant, termed CK1δ NP, in which all the serine and threonine residues in the tail were mutated to alanine (Fig 3A). Phosphorylation of CK1δ *in vivo* is actively reversed by serine-threonine phosphatases that can be inhibited by phosphatase inhibitors [20,21]. While the wild-type CK1δ (CK1δ WT) showed the expected electrophoretic mobility shift after a 30 minute exposure to 40 nM of the cell permeable serine/threonine phosphatase inhibitor Calyculin A, CK1δ NP did not exhibit the same mobility shift, suggesting that this mutant has decreased phosphorylation sites (Fig 3B).

To investigate the activity of the CK1δ NP mutant, an IP kinase assay was utilized, using dephosphorylated casein as a substrate. ^32^P-ATP incorporation was seen both in CK1δ itself due to auto-phosphorylation, as well as in casein (Fig 3C). CK1δ NP exhibited significantly lower autophosphorylation and phosphorylation of casein (Fig 3C, 3D and 3E). This is in agreement with our hypothesis that CK1δ is more active in a hypophosphorylated state.

To investigate the effect of auto-phosphorylation on inhibition of mutant CK1δ activity, we first incubated the immunoprecipitated CK1δ with ^32^P-ATP in the absence of casein. Auto-phosphorylation was allowed to occur before casein was added 15 minutes later. While the autophosphorylation pattern remains the same, CK1δ WT activity against casein was reduced by autophosphorylation, whereas CK1δ NP activity against casein was unchanged (Fig 3C, 3D and 3E). These data show that CK1δ NP is more active than CK1δ WT *in vitro* and is not significantly inhibited by autophosphorylation.

### Non-phosphorylatable mutants of CK1δ, including the single mutant S347A, exhibited higher specific activity on PER2

**Fig 4:**
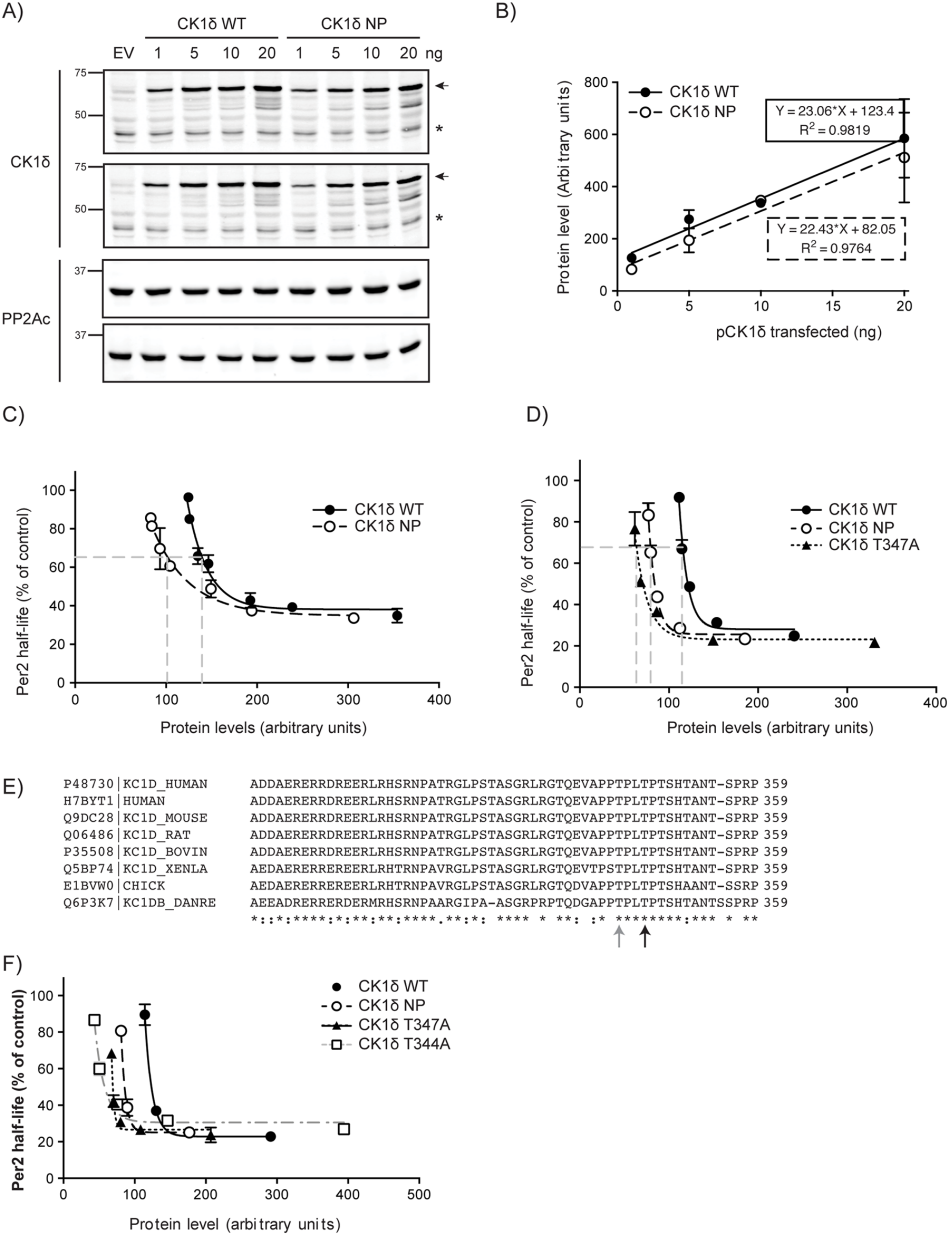
CK1δ NP increases Per2 degradation. **A, B) Protein expression standard curve**. The indicated amounts of 6Myc-CK1δ wild-type and NP expression plasmids were transiently transfected into HEK293 cells and protein abundance assessed by SDS-PAGE and immunoblotting with anti-CK1δ Mab. PP2Ac was used as load control. Duplicate experiments are shown. Arrows indicate Myc-tagged CK1δ while asterisks indicate endogenous CK1δ. B) CK1δ protein abundance in (A) was quantified by densitometry and standard curves of the different expression plasmids established. **C) Non-phosphorylatable (NP) CK1δ accelerates PER2 degradation.** Increasing amounts of wild-type and NP CK1δ were co-expressed with S659A PER2::LUC in HEK293 cells and PER2 half-life determined as above. Per2 half-life was plotted against Myc-CK1δ protein abundance. Because PER2 half-life plateaus at 30% of control, the half maximal decrease occurs at 65% of control. **D) Mutation of CK1δ T347A phenocopies the NP mutant.** Increasing amounts of wild-type and mutant CK1δ expression plasmids were transiently co-transfected with 10 ng PER2::LUC into HEK293 cells, and analyzed as in (C). **E) Multiple sequence alignment of CK1δ at the T347 region.** T347 (black arrow) is 3 residues downstream of a phosphorylation site T344 (grey arrow), suggestive of a CK1 specific phosphorylation site. **F) T344A mutant phenocopies both the NP and T347A mutants, and accelerates PER2 degradation.** Data shown are average from three independent experiments done in duplicates.

We next tested if CK1δ NP was more active on PER2. CK1δ WT and NP were co-expressed with PER2(S659A)::LUC, and PER2 stability was measured. We noticed that the *CK1δ NP* plasmid expressed less protein than WT (Figs 3B and 4A), probably as a consequence of the multiple amino acid substitutions. As such, we also took protein abundance into consideration when assessing CK1δ activity. Thus, a standard curve for CK1δ expression was established (Fig 4B). When protein abundance was plotted against PER2 half-life, we found that less CK1δ NP was required to bring about a half-maximal decrease in PER2 half-life (Fig 4C).

To determine the specific phosphorylation site(s) on CK1δ responsible for the increased kinase activity, we screened a series of individual phosphorylation site mutants, based on the information at Phosphosite. We found that T347A mutation could phenocopy the effect of the CK1δ NP mutant (Fig 4D). Given CK1’s preferred phosphorylation motif of pS/T-X-X-S/T, we examined if T344 also regulated CK1δ activity (Fig 4E). Interestingly, CK1δ T344A also phenocopied the effect of the CK1δ NP mutant (Fig 4F).

### Characterization of CK1δ T347 phosphorylation

**Fig 5:**
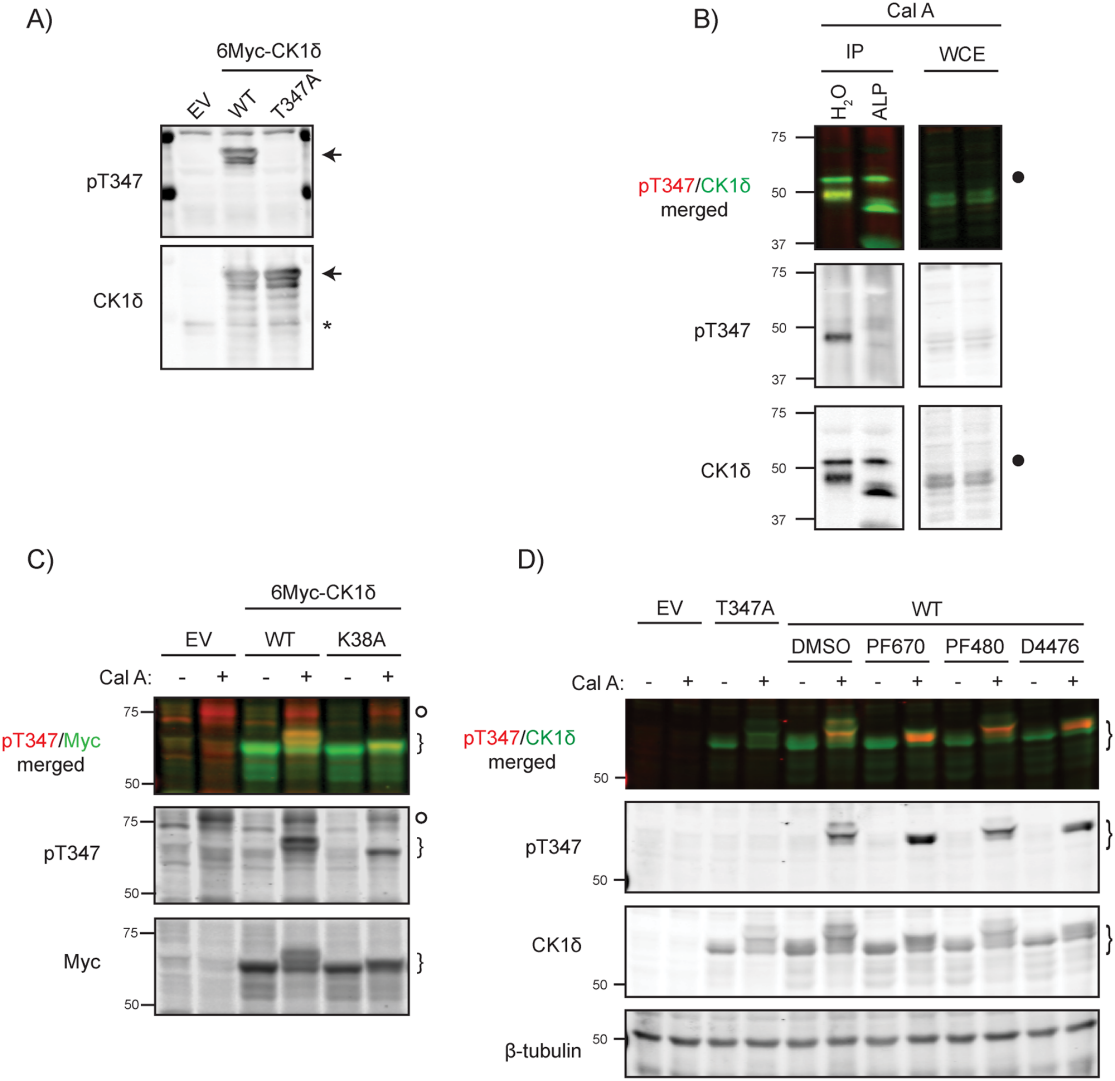
In vivo, CK1δ T347 is phosphorylated in *trans*. **A) Characterization of a specific CK1δ pT347 phospho-epitope antibody.** HEK293 cells over-expressing wild-type or T347A 6Myc-CK1δ were treated with 40 nM calyculin A for 30 minutes prior to lysis and immunoblotting with anti-pT347 or anti-CK1δ antibodies. Arrows indicate Myc-tagged CK1δ while asterisks indicate endogenous CK1δ, which also serves as a load control. **B) Phosphorylation of T347 on endogenous CK1δ is reversed by alkaline phosphatase.** Endogenous CK1δ was immunoprecipitated from calyculin A-treated HEK293 cells. Pelleted beads were treated with either vehicle or alkaline phosphatase (ALP) prior to analysis by SDS-PAGE and immunoblotting with the indicated antibodies. Calyculin-enhanced CK1δ phosphorylation causes a marked electrophoretic mobility shift that is reversed by alkaline phosphatase. Closed circle denotes the IgG heavy chain present in the immunoprecipitate. WCE denotes whole cell extract. **C) T347 is not a CK1δ autophosphorylation site.** Wild-type or catalytically-inactive K38A 6Myc-CK1δ were transiently expressed in HEK293 cells. Following a 30 minute treatment with either DMSO or 40 nM calyculin A, cells were lysed and immunoblotted for anti-pT347 or anti-CK1δ antibodies. Kinase-dead CK1δ has a much reduced calyculin-induced electrophoretic mobility shift but is still phosphorylated on T347. Myc-tagged CK1δ indicated by curly brackets. Open circle denotes a Calyculin A dependent non-specific band. **D) Phosphorylation of CK1δ at T347 is not dependent on CK1 activity.** HEK293 cells over-expressing wild-type CK1δ were treated with CK1 inhibitors PF670462 (PF670, 1 μM), PF4800567 (PF480, 1 μM), or D4476 (30 μM) for 3 hours, followed by addition for 30 min of either DMSO or 40 nM Calyculin A. Cell lysates were immunoblotted for anti-pT347 or anti-Myc antibodies. CK1 inhibitors block the autophosphorylation-induced mobility shift but do not decrease phosphorylation of T347. Myc-tagged CK1δ indicated by curly brackets.

In order to study T347 phosphorylation, we generated a phospho-specific antibody against CK1δ pT347. Confirming the specificity of the phospho-epitope specific antibody, when CK1δ was overexpressed in the presence of Calyculin A, the antibody detected phospho-CK1δ in wild-type but not T347A CK1δ (Fig 5A). Calyculin A induced phosphorylation of T347 in endogenous CK1δ was detectable in immunoprecipitates of CK1δ but not in whole cell lysates (Fig 5B). Treatment of immunoprecipitated CK1δ with alkaline phosphatase increased the electrophoretic mobility of CK1δ, confirming the activity of the phosphatase, and eliminated immunoreactivity with the phosphoT347 antibody, further confirming the specificity of the antibody.

Based on the finding that autophosphorylation resulted in inhibition of CK1δ kinase activity, we initially presumed that the inhibitor phosphorylation sites in the CK1δ tail were the result of autophosphorylation. To test that assumption, kinase-dead CK1δ was transiently overexpressed. Unexpectedly, catalytically inactive CK1δ K38A was still phosphorylated on T347 in the presence of Calyculin A (Fig 5C). The K38A mutant’s inability to autophosphorylate is evident in the lack of mobility shift after the addition of Calyculin A. Thus, phosphorylation of T347 is not due to an intramolecular auto-phosphorylation. However, in view of the phosphorylation sites arrangement matching the CK1 phosphorylation motif, we speculated that the T347 phosphorylation might be occurring in trans, phosphorylated by either endogenous CK1δ or other CK1 isoforms. To test this, cells overexpressing Myc-tagged CK1δ were treated with either the pan-CK1δ/CK1ε inhibitor PF670462, CK1ε specific inhibitor PF4800567, both inhibitors together, or the pan CK1α, δ and ε inhibitor D4476, all with or without Calyculin A treatment (Fig 5D). Enhanced T347 phosphorylation in the presence of Calyculin A was seen regardless of the CK1 inhibitor added, while the activity of PF670462 and D4476 was confirmed by their effect on CK1δ electrophoretic mobility. (Since PF4800567 is a CK1ε specific inhibitor, no effect on CK1δ autophosphorylation was observed.) Thus, in vivo phosphorylation of CK1δ T347 is not dependent on CK1α, δ or ε.

### Cdk are putative kinases phosphorylating T347

**Fig 6:**
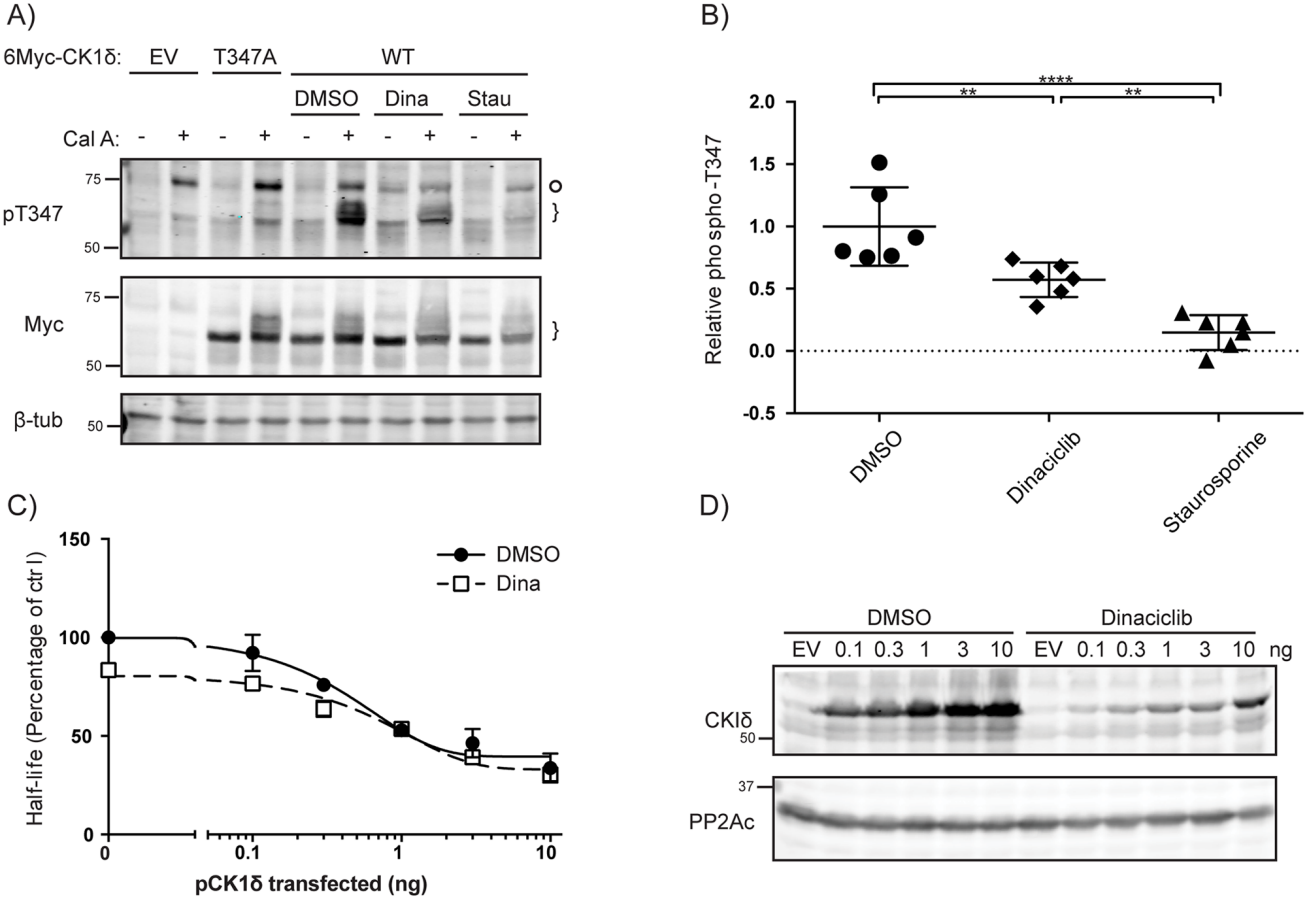
Dinaciclib inhibits CK1δ pT347 phosphorylation and increases CK1δ activity against Per2. **A, B) Diverse kinase inhibitors decrease CK1δ T347phosphorylation in cells.** HEK293 cells expressing myc-CK1δ were treated with DMSO, 100 nM Dinaciclib, 5 μM Staurosporine or 10 μM SB203580 for 6 hours. Prior to lysis, DMSO or 40 nM Calyculin A was then added for 30 minutes. Lysates were immunoblotted with anti-pT347 or anti-Myc. B) Phosphorylation on T347 was quantified from (A) using densitometry. **C, D) Dinaciclib accelerates S659A Per2 degradation.** 10 ng S659A Per2:luc was co-transfected with indicated quantity of pCK1δ into HEK293 cells. 20 hours post-transfection, cells were treated with DMSO or 10 nM dinaciclib. 2 hours post-treatment, 40 μg/ml cycloheximide was added. Degradation of PER2 was calculated by measuring luciferase activity. D) Expression of pCK1δ in (C) after 72 hours Dinaciclib treatment was confirmed by western blot analysis.

Our data on the role of phosphoT347 indicates changes in the phosphorylation of this site regulate circadian rhythms. Inspection of the sequence context of T344 and T347 (Fig 4E) suggested they could be targeted by proline-directed kinases. Indeed, the kinase prediction database Scansite [35] predicted Cdk1 and Cdk5 as potential kinases for T347, a prediction confirmed by Ianes et al. while our studies were ongoing [23]. Consistent with this, we find that 100 nM dinaciclib, a pan-Cdk inhibitor targeting Cdks 1, 2, 5 and 9 [36], reduced (but did not completely inhibit) phosphorylation of CK1δ T347 (Fig 6A and 6B). This finding is in agreement with recent work [23] showing CK1δ can phosphorylated by Cdk2 and Cdk5. However, the presence of residual phosphorylation in the presence of 100 nM dinaciclib, which has an IC50 of 1–4 nM[36], suggests that other proline directed kinase(s) may also be capable of phosphorylating T347. Indeed, 10 μM staurosporine, a pan-kinase inhibitor, completely abrogated the phosphorylation of CK1δ on T347. Ianes et al. recently reported that CDK activity decreased CK1δ activity in vitro, and inhibition of CDKs by dinaciclib increased CK1δ’s activity in pancreatic cancer cells [23]. Consistent with this, in our system dinaciclib reduced PER2 half-life in the presence of co-expressed CK1δ, especially at the lower doses of CK1δ (Fig 6C). However, dinaciclib also marked reduced the expression of exogenous CK1δ (Fig 6D), complicating further analysis.

## Discussion

Period protein degradation in the accumulation phase of the circadian cycle is regulated by CK1δ/ε. The critical importance of CK1 activity in circadian rhythms is highlighted by the number of sporadic mutations in mammalian CK1δ, CK1ε, and CK1 phosphorylation sites that alter period [17,37–39]. CK1 phosphorylates PER2 in two sites, the FASP site and the β-TrCP site. Phosphorylation of the FASP site inhibits phosphorylation of the β-TrCP site, creating a temperature sensitive phosphoswitch [11,17]. Here, we show that the activity of CK1δ against the β-TrCP site can also be regulated by changes in the phosphorylation of CK1δ at a specific set of sites, T344 and T347, in the CK1δ regulatory domain. The kinase(s) responsible for in vivo phosphorylation of CK1δ T344/347 are thus poised to influence CK1δ activity and hence PER2 degradation. Our data suggests that dinaciclib-sensitive CDKs may be these kinases, but that other proline-directed kinases such as ERK1 and p38 may also phosphorylate these sites. The broad anti-proliferative activity of these CDK inhibitors in transformed cells precluded our testing their effect on circadian rhythms in culture. However, consistent with our findings, others have previously reported CDK inhibitors impacted the circadian rhythms of both liver explants and mollusks [40,41].

The effect of proline directed kinases such as the cyclin-dependent kinases on CK1δ activity and hence on PER2 stability suggests a mechanism for cell cycle signaling events to influence circadian period, as has been recently suggested [42]. The concept of a crosstalk between the cell cycle and the circadian rhythms is not new. Initial studies, however, found that the circadian period gates the cell cycle, rather than the cell cycle altering the circadian period. For example, studies done in *Euglena gracilis* showed synchronization of cell division by illumination [43]. It was proposed that the gating of cell division by the biological clock protected cells from DNA damage by daytime solar UV radiation. In the regenerating mouse liver, entry into the cell cycle is gated by circadian rhythms as well [44]. Our results are consistent with recent findings that suggest a signal can come in the other direction, that is, that cell cycle or other signaling events can influence CK1δ activity, PER2 stability, and hence circadian period [42]. The potential involvement of MAP kinases also suggests a mechanism for growth factor signaling to impact on CK1δ activity.

Several external events can regulate circadian clocks and could function through changes in CK1 activity. Signals from the retinohypothalamic tract impinge on SCN and other neurons to alter period in response to changing light conditions. It is relevant here that metabotropic glutamate receptor signaling can regulate CK1 activity via phosphorylation[26]. Cells in culture can be circadian-synchronized by a variety of stimuli including media change, serum shock, and glucocorticoids [45]. These events activate signaling pathways that could work in part through changes in CK1δ phosphorylation. Chung and co-workers reported that activation of AMPK by metformin led to phase advance by phosphorylation of CK1ε at S389 and increased phosphorylation and degradation of PER2 [46]. Our laboratory demonstrated that Wnt signaling can regulate CK1ε through changes in the phosphorylation of its regulatory domain [29]. Consistent with a role for a Wnt-CK1 axis in circadian rhythms, Hong and co-workers found an interaction between Wnt signaling and circadian rhythms in mouse intestinal organoid culture [1].

In summary, we describe here a specific mechanism for the regulation of CK1δ activity by proline-directed kinases, and demonstrate the relevance of this regulation in the control of a key CK1δ substrate, the PER2 protein. Given the importance of CK1δ and CK1ε in diverse signaling pathways, these findings in addition have a broader impact on our understanding of how these essential regulators are regulated.
